# 

**Published:** 1865-04

**Authors:** 


Volume XXII. Number 4.

### THE CHICAGO MEDICAL JOURNAL

DE LASKIE MILLER, M. D.,

EFHRAIM INGALS, M. D.,

Editors and Proprietors.

APRIL, 1865.

CHICAGO:
J. C. W. BAILEY, BOOK AND JOB PRINTER, 130 CLARK STREET.
1865.

